# Construction of high-quality genomes and gene catalogue for culturable microbes of sugarcane (*Saccharum* spp.)

**DOI:** 10.1038/s41597-024-03379-w

**Published:** 2024-05-24

**Authors:** Liang Wu, Haidong Lin, Lijun zhang, Ta Quang Kiet, Peng Liu, Jinkang Song, Yong Duan, Chunyu Hu, Hao Yang, Weixing Duan, Xiping Yang

**Affiliations:** 1 State Key Laboratory for Conservation and Utilization of Subtropical Agro-Bioresources, Guangxi University, Nanning, 530005 China; 2 National Key Laboratory for Biological Breeding of Tropical Crops, Kunming, 650221 China; 3 Sugarcane Research Institute, Guangxi Academy of Agricultural Sciences / Sugarcane Research Center, Chinese Academy of Agricultural Sciences / Guangxi Key Laboratory of Sugarcane Genetic Improvement / Key Laboratory of Sugarcane Biotechnology and Genetic Improvement (Guangxi), Ministry of Agriculture and Rural Affairs, Nanning, Guangxi 530007 China

**Keywords:** Metagenomics, Metagenomics

## Abstract

Microbes living inside or around sugarcane (*Saccharum* spp.) are crucial for their resistance to abiotic and biotic stress, growth, and development. Sequences of microbial genomes and genes are helpful to understand the function of these microbes. However, there is currently a lack of such knowledge in sugarcane. Here, we combined Nanopore and Illumina sequencing technologies to successfully construct the first high-quality metagenome-assembled genomes (MAGs) and gene catalogues of sugarcane culturable microbes (GCSCMs), which contained 175 species-level genome bins (SGBs), and 7,771,501 non-redundant genes. The SGBs included 79 novel culturable bacteria genomes, and 3 bacterial genomes with nitrogen-fixing gene clusters. Four single scaffold near-complete circular MAGs (cMAGs) with 0% contamination were obtained from Nanopore sequencing data. In conclusion, we have filled a research gap in the genomes and gene catalogues of culturable microbes of sugarcane, providing a vital data resource for further understanding the genetic basis and functions of these microbes. In addition, our methodology and results can provide guidance and reference for other plant microbial genome and gene catalogue studies.

## Background & Summary

Sugarcane (*Saccharum* spp.) is an important cash crop, whose stalks are rich in sugar, and are widely used in food, energy production, and industrial raw materials^1^. Globally, sugarcane has been planted over 27 million hectares, and is commonly grown in 120 countries and regions^2^. In recent years, the increase in sugarcane yields has been stagnant due to the excessive use of chemical fertilizers^3^, soil acidification^4,5^, pests^6^ and diseases^7^ in sugarcane cultivation. How to improve the yield has become a current research hotspot in the field of sugarcane agricultural research. Endophytes and rhizosphere soil microbes, the two primary forms of microbiota, are crucial for fostering plant growth, development and tolerance to stresses^8,9^. Hence, it is imperative to investigate the community composition and potential functions of sugarcane endophytes and its rhizosphere soil microbes, to screen functional strains that are beneficial to sugarcane yield enhancement, and to develop and utilize applicable bacterial resources for the sustainable development of the sugarcane industry.

Endophytes are microbes that live inside plant tissues (leaves, stems, roots), and may form a symbiotic relationship with plant^10^. These microbes can promote plant growth and development by fixing nitrogen and providing growth-regulating substances^11^. Since the initial isolation of endophytes from sugarcane by Dobereiner in 1961^12,13^, several endophytic flora from 21 genera, including *Bacillus*, *Burkholderia*, *Enterobacteriaceae*, and *Pantoea*, have been characterized from sugarcane tissues (stems and leaves)^14–17^. Many culturable bacteria have been isolated from sugarcane’s rhizosphere soil and roots, including *Azospirillum*, *Burkholderia*, *Klebsiella, Enterobacter* and *Erwinia*^18–21^. Due to limits of culture conditions, in the context of traditional colony culture and single bacteria isolation procedures, it is typical to result in a significant reduction in the number of microbial species detected in the sample^22–24^. Furthermore, isolating and identifying individual microbe necessitates a substantial allocation of both human and material resources. Thus, in this research, we employed a technique to culture microbes from sugarcane’s inner tissues and rhizosphere soil by using multiple plant genotypes and media, which is able to enhance the cultivation of microbes.

In recent years, microbial genomics research has experienced significant advancement due to the ongoing progress in high-throughput sequencing technology^25,26^. These technologies, such as Nanopore and Illumina sequencing, have proven effective in acquiring comprehensive genomic and gene sequence data of microbial populations^27,28^, and have greatly facilitated the investigation of microbes’ genetic makeup and functional characteristics. The construction of reference genomes and gene catalogues of microbes for the global oceans^29^, human^25,30^, soil^31,32^, and animal gut^33–35^ has been completed. However, there are few applications and reports on the construction of genomes and gene catalogues using plant microbiota metagenomes, and there need to be more research on sugarcane microbiota. Therefore, constructing a complete genome and gene catalogue of culturable microbial species of sugarcane is necessary for studying sugarcane microbiota.

**Fig. 1:**
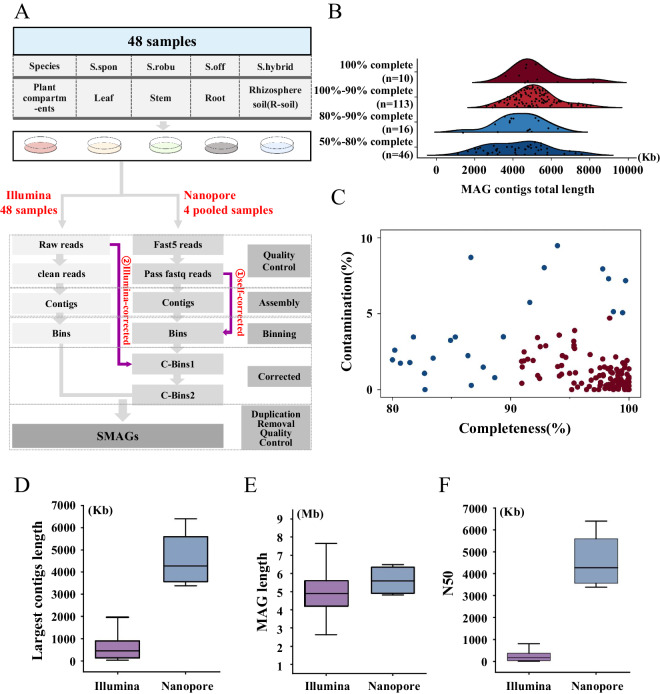
Metagenome-assembled genomes (MAGs) construction and assembly process. (**A**) Schematic diagram of constructing MAGs using Nanopore and Illumina sequencing data. S. spon, S. robu, S. off, and S. hybrid stood for *S. spontaneum*, *S. robustum*, *S. officinarum* and *Saccharum* hybrid cultivar, respectively. Three genotypes for each species were included for culture of microbes (see Supplementary Data 1 for detail). Five media were used, including NA (Nutrient Agar), AM (Ashby’s Medium), BM (Burke’s Medium), R2A (R2A Medium), and PDA (Potato Dextrose Agar). C-Bins1 represented bins generated in the first round of correction, and C-Bins2 represented bins generated in the second round of correction. (**B**) Distribution of genome integrity and distribution of quality classes of MAGs. Horizontal coordinates denoted MAG length, vertical coordinates denoted MAG integrity, and n represented the number of MAGs. (**C**) Distribution of integrity and contamination of high-quality MAGs. Horizontal coordinates indicated MAG completeness, and vertical coordinates indicated MAG contamination. (**D**–**F**) Comparison of three critical metrics of assembly results using Nanopore and Illumina sequence data, with vertical coordinates indicating the length of the most extended overlapping group, MAG length, and N50 value, respectively.

To cover this void, we sequenced 48 samples (mixtures of culturable microbes) from multiple plant compartments, genotypes, and sugarcane species by Nanopore sequencing and Illumina sequencing (Fig. 1A). Through this study, we have the following findings: (1) Constructed a non-redundant gene catalogue (GCSCMs)^36^ of culturable microbes in sugarcane containing 7,771,501 genes; (2) Assembled 175 species-level genome bins (SGBs)^37,38^ at the species level by the metagenome assembly technique, which included 77 potentially novel culturable bacterial species; (3) Successfully assembled single scaffold circular genomes^37,38^ from Nanopore Long Reads (LRs) with 0% contamination and near-complete. Thus, the utilization of Nanopore and Illumina sequencing technologies in constructing the genome and gene catalogue of the sugarcane culturable microbiome held the potential to enhance our comprehension of the characteristics of this microbiome. In summary, the results provide substantial genomes and gene resources of endophytes and rhizosphere soil microbial resources in sugarcane to explore sugarcane-microbe interaction and microbial functions.

## Methods

### Sample collection, isolation and culture of microbes

In order to ensure the diversity of samples, three species and one hybrid of sugarcane (*S. spontaneum*, *S. robustum*, *S. officinarum*, and *S*. hybrid) with three genotypes from each were chosen for this study (Fig. 1A, Supplementary Data 1). These materials included 48 samples for microbial isolation and cultivation, including leaves (the first leaf of the sugarcane plant that is fully green from the bottom to the top), stems (taken from the second node above the ground), and roots (the soil still adhering to the roots was collected as rhizosphere soil samples by vigorous shaking). The samples were immediately maintained in sterile bags and appropriately kept in an incubator set at 4 °C. Within 24 hours of sample collection, culturable microbes from sugarcane were isolated and cultured.

For the purpose of isolating rhizosphere soil microbes, 5 g of roots retaining small particles of soil attached need to be taken, and 100 ml of sterile water added. Then, it was washed using ultrasonic oscillation (Model: KQ-600E, Frequency: 28KHZ) for 1 min, and the step was repeated 2 times. After the oscillatory washing, the sample was left to stand for 10 min in order to isolate the rhizosphere soil microbes. The treatment of leaves, stems, and roots required surface sterilization. Leaves, stems, and roots (5 g after oscillatory washing as stated above) were soaked in 75% alcohol for 3 min and rinsed with sterile water 5 times after soaking. Then, the leaves, stems, and roots were immersed in 3% sodium hypochlorite for 7, 5, and 3 min, respectively, before being rinsed five times with sterile water. For the isolation of endophytes from leaves, stems, and roots, the processed samples were clamped into the sterile mortar with tweezers and fully ground by adding 100 ml of sterile water. After grinding, it was left to stand for 10 min in horizontal flow clean bench (SW-CJ-1CU). The four supernatants obtained above were the original bacterial suspension. Except for the 10-fold dilution of the original bacterial solution needed for rhizosphere soil, the original bacterial suspension was used for the cultivation of culturable microbes. Next, 100 μl was aspirated and spread separately on five kinds of solid media, namely Nutrient Agar, Ashby’s Medium, Burke’s Medium, R2A Medium, and Potato Dextrose Agar (Table 1). After spreading evenly by the rollerball method, the plates were inverted and incubated at 28 °C for 72 h.

**Table 1:** Cultural medium and composition.

Cultural medium	Composition
Nutrient Agar	Peptone 10 g/L 、Beef extract 3 g/L、NaCl 5 g/L Agar 20 g/L、pH: 7.0-7.2.
Ashby’s Medium	Mannitol 10 g/L、CaSO_4_.2H_2_O 0.2 g/L、KH_2_PO_4_ 0.2 g/L, MgSO_4_.7H_2_O 0.2 g/L、CaCO_3_ 5 g/L、NaCl 0.2 g/L、Agar 20 g/L、pH: 6.8-7.0.
Burke’s Medium	MgSO_4_ 0.2 g/L、KH_2_PO_4_ 0.2 g/L、CaSO_4_ 0.13 g/L、Fecl_3_ 1.45 mg/L、MoNa_2_O_4_ 0.253 mg/L、Sugar 20 g/L、Agar 20 g/L、pH: 7.2-7.3.
R2A Medium	Yeast power 0.5 g/L、Peptone 0.5 g/L、Casamino acids 0.5 g/L、Glucose
	0.5 g/L、Soluble starch 0.5 g/L、Sodium pyruvate 0.3 g/L、K_2_HPO_4_ 0.3 g/L、
	MgSO_4_ 0.05 g/L、Agar 20 g/L、pH: 6.5-7.0.
Potato Dextrose Agar	Potato 200 g/L、Glucose 20 g/L、Agar 20 g/L、H_2_O 1000 ml、PH:7.4-7.6.

*Note: Nutrient Agar is a simple and effective medium for the cultivation and experimentation of a wide range of microorganisms and is one of the most commonly used media in the field of microbiology. **Ashby’s Medium** is mainly used for culture and screening of rhizobia. **Burk’s Medium** is used for isolation and cultivation of nitrogen fixing bacteria such as Azotobacter species from soil. **R2A Medium** is widely used to isolate and culture microorganisms from environmental samples (soil, water, air). **Potato Dextrose Agar** is a commonly used fungal medium for the growth and propagation of a wide range of fungi.

### DNA extraction and quality control

**Fig. 2:**
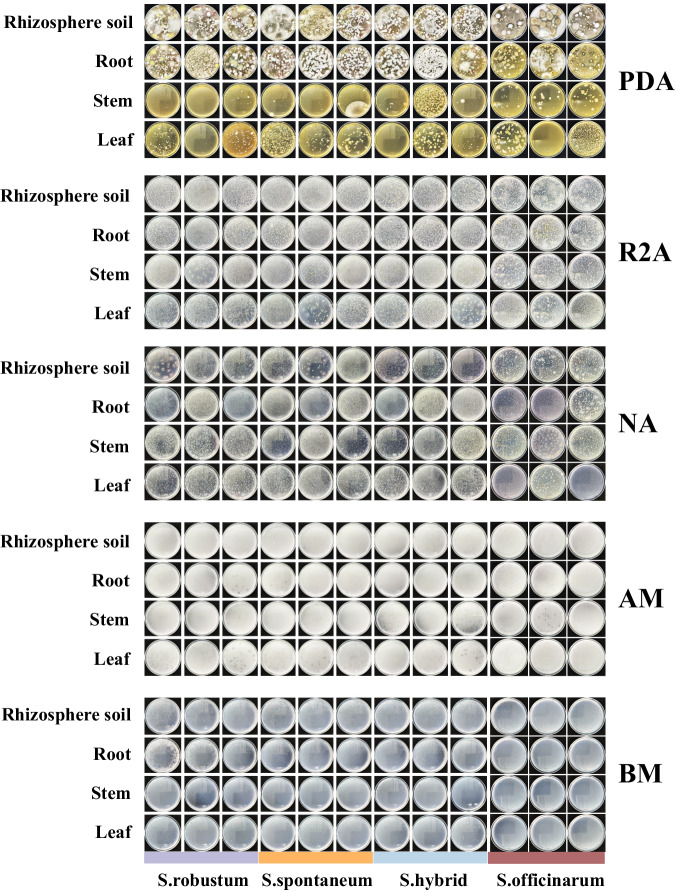
Growth of sugarcane culturable microbes after 72 h. Horizontal coordinates indicated the three sugarcane species and a hybrid (each with three genotypes), and vertical coordinated indicate the plant compartments for the isolates. PDA (Potato Dextrose Agar), R2A (R2A Medium), NA (Nutrient Agar), AM (Ashby’s Medium), and BM (Burke’s Medium) denoted five different media.

Following a 72-hour incubation to ensure the growth of the culturable microbes (Fig. 2), we conducted whole-genome metagenome sequencing on the genomic DNA extracted.1 ml of sterile water was aspirated as a rinse solution using a sterile pipette in horizontal flow clean bench. Rinsing was repeated 4 times, and the rinsed solution was aspirated into a 50 ml centrifuge tube. In order to collect all the colonies on the five solid media, rinses were repeated four times, and the rinses from the same bacterial suspension were aspirated into a 50 ml centrifuge tube. A total of 48 rinses of culturable microbes from different plant compartments and different genotypes of sugarcane were obtained. After shaking and mixing, 2 ml of the rinse suspension was pipetted into 2 ml centrifuge tubes and centrifuged separately, the supernatant was discarded, and the precipitate was kept at −80 °C immediately until the microbial DNA extraction for Illumina data sequencing. Meanwhile, 3 ml of the rinses from the same sugarcane species (3 genotypes, 4 plant compartments) were aspirated into the same 50 ml centrifuge tube and centrifuged. The supernatant was discarded, and the precipitate was immediately kept at −80 °C until the microbial DNA extraction for sequencing.

To isolate high-quality DNA of culturable culturable microbes from sugarcane, we used the CTAB/NaCl method for DNA extraction^39^. The integrity of the DNA was tested by capillary electrophoresis using a fragment analyser (AATI) with the use of a Qubit^®^ 2.0 fluorometer and a Nanodrop kit for precise quantification and purity determination. The quality of the extracted DNA was assessed using 1% agarose gel electrophoresis.

### Library construction and sequencing

To create Illumina sequencing libraries, we combined the NEBNext® UltraTM DNA Library Preparation Kit (New England Biolabs, USA) with 1 μg of DNA. Index codes were appended to the sequencing primers. The ends of the isolated DNA were repaired after being sonicated into 350 bp fragments. Following end repair, an adenine was added to the 3’ ends of the DNA fragments, and then adaptor sequences were ligated to both ends of the A-tailed DNA. These libraries were cleaned using Beckman Coulter’s AMPure XP technology in Brea, California, USA. With the use of an Agilent 2100 Bioanalyzer and real-time fluorescence quantitative PCR, the purified products were examined for size distribution and quantity. All samples were subjected to paired-end sequencing utilizing an Illumina NovaSeq 6000 platform with a read length of 150 base pairs (PE150) once the library’s quality had been confirmed.

According to the manufacturer’s instructions, we used 2.5 μg of extracted DNA for library preparation using the SQK-LSK110 Ligation Sequencing Kit (Oxford Nanopore Technologies, Oxford, UK) to create PromethION libraries. A Megaruptor (Diagenode, NJ, USA) was used to process the DNA, and BluePippin was used to screen for DNA fragments longer than 10 Kb. The lengths of the repaired pieces were verified, and specific barcodes and poly(A)tail were inserted. After mixing samples with various barcodes in equimolar proportions and purifying them, the DNA libraries were ready. A Qubit fluorometer was used to measure the DNA concentration. Utilizing Nanopore sequencing technology (Oxford Nanopore PromethION Sequencer: PromethION P48), sequencing was carried out after ensuring the libraries’ quality.

### Data quality control

Low-quality base pairs, and reads shorter than 150 bp of raw data obtained from the Illumina sequencing platform were removed using fastp (v0.19.4 https://github.com/OpenGene/fastp)^40^. Parameters were set to “--cut_by_quality3 -W 4 -M 20 -n 5 -c -l 50 -w 3”. The Nanopore reads fast5 data were converted to fastq format using Guppy (v.3.03 https://community.nanoporetech.com) for Nanopore sequencing data. Then, NanoPlot (ver.1.18.2 https://github.com/wdecoster/NanoPlot)^41^ was used to perform quality control on the fastq format data by filtering low-quality sequences, splice sequences, and etc. The threshold value of the filtering criterion was set to meanQ > 7 to remove low-quality sequences, and the parameters were set to ‘-t 20,--loglength,--N50’. After quality control, the clean and high-quality reads obtained from both sequencing methods were used for further in-depth analyses.

Illumina sequencing yielded a total of 1.1 Tb of Illumina sequencing data^42^. After quality control of the sequencing data, 1.08 Tb of clean, high-quality pair-ended data were retained for further analysis, and an average of 22.50 Gb/sample was attained (Supplementary Data 1). Nanopore sequencing is commonly employed to generate long reads, which are then utilized to assemble circular metagenome-assembled genomes (cMAGs). To improve the quality of metagenome assembly, we mixed 48 samples into four pooled samples by sugarcane species (Supplementary Data 2) for Nanopore sequencing. We obtained 61.35 Gb of clean long reads sequencing data^42^ (65.63 Gb of raw data, with data efficiency > 93.48%) with an average read length of 8.01 Kb (Supplementary Data 2).

### Metagenomics assembly and binning

To obtain comprehensive genomic data, a single-sample *de novo* assembly of quality-controlled Illumina sequencing data was performed using MetaSPAdes (v.3.13.0 https://github.com/ablab/spades)^43^ with default K-mer parameters. Only contigs of ≥ 1000 bp were retained. A total of 13,481,007 contigs were obtained with a minimum length of 1000 bp. The quality-controlled Illumina sequencing data were mapped to the contigs using BWA MEM (v.0.7.17 https://github.com/lh3/bwa)^44^ to produce Sam format files containing the comparison information. Samtools (v.1.10 https://github.com/samtools/samtools/releases)^45^ was used to convert the Sam files to Bam format. Sequencing depths of contigs were generated from Bam files using the script jgi_summarise_BAM_contig_depth that comes with MetaBAT2 (v.2.12.1 https://github.com/bioboxes/metaBAT)^46^ Based on the sequence characteristics and sequencing depth of these contigs, a total of 1485 bins were generated from 13,481,007 contigs using MetaBAT2.

For Nanopore sequencing data, the four Nanopore datasets were individually *de novo* assembled using metaFlye (v.2.8.3 https://github.com/fenderglass/Flye)^47^ after quality control. The Nanopore reads were mapped onto the contigs generated from the Nanopore sequencing data by minimap2 (v.2.22 https://github.com/lh3/minimap2)^48^ to produce Sam format files containing comparison information. The next binning steps were consistent with the processing of Illumina sequencing data. In total, 62 bins were generated from the Nanopore sequencing data. To improve the reliability of binning, we performed two rounds of correction. The first round of correction was a self-correction, where Nanopore reads were rearranged onto contigs by Medaka (v0.6.5 https://github.com/nanoporetech/medaka) to obtain consensus sequences. Then, the second round of correction was performed, using Pilon (v.1.12 https://github.com/broadinstitute/pilon)^49^ to map Illumina reads onto the consensus sequence based on the Pilon correction of BWA-MEM (v.0.7.17) to correct Indel errors.

A total of 1547 bins were generated from Illumina reads and Nanopore reads.The Illumina sequencing data produced bins with a maximum length of 23.79 Mb and a maximum N50 value of 2.97 Mb, and the Nanopore sequencing data produced bins with a maximum length of 11.78 Mb and a maximum N50 value of 2.30 Mb (Supplementary Data 3). 717 MAGs were generated by removing duplications of all bins usingdRep (v.1.1.2 https://github.com/MrOlm/drep)^50^ at 99% ANI (equivalent to strain level), with 681 and 36 bins from Illumina sequencing and Nanopore sequencing data, respectively (Supplementary Data 4). The completeness and contamination of the above 717 MAGs were estimated based on the lineage_wf workflow using CheckM (v.1.0.7 https://github.com/Ecogenomics/CheckM)^51^. After quality assessment, no complete eukaryotic and viral genomes were found in the obtained non-redundant metagenome-assembled genomes (MAGs). Subsequently, we focused on the analysis of prokaryotic MAGs, and obtained a total of 185 metagenome-assembled genomes (SMAGs) of culturable bacteria from sugarcane (Supplementary Data 5). According to the “Minimum Information about Metagenome Assembled Genomes (MIMAG)” standard^52^, all of these assembled SMAGs met or exceeded the standard of medium quality (defined as > 50% completeness and < 10% contamination)^53^ (Fig. 1B). Of these, 171 SMAGs were from Illumina sequencing, and 14 SMAGs were from Nanopore sequencing. Among the 185 SMAGs, 139 had high-quality genomes (defined as > 80% completeness and 10% contamination) (Fig. 1C), including 95 with > 95% completeness and < 5% contamination, 12 with > 95% completeness and 0% contamination, and 4 with 100% completeness and 0% contamination (Supplementary Data 5). The Nanopore data outperformed Illumina data in assembly length, N50 value, and overlap cluster length for high-quality SMAGs (Fig. 1D).

### Phylogenetic analysis and annotation of SMAGs

To determine the phylogenetic affiliation and diversity of the 185 SMAGs,we used the “classify_wf” workflow in GTDB-TK (v.0.3.0; default settings http://gtdb.ecogenomic.org/)^54^ to identify 120 bacterial marker genes and constructed multiple sequence pairs based on these marker genes. The generated multiple sequence comparison FASTA files were subjected to maximum likelihood phylogenetic tree inference using IQ-TREE. Final visualization was performed using the ChiPlot web tool (https://www.chiplot.online/).

**Fig. 3:**
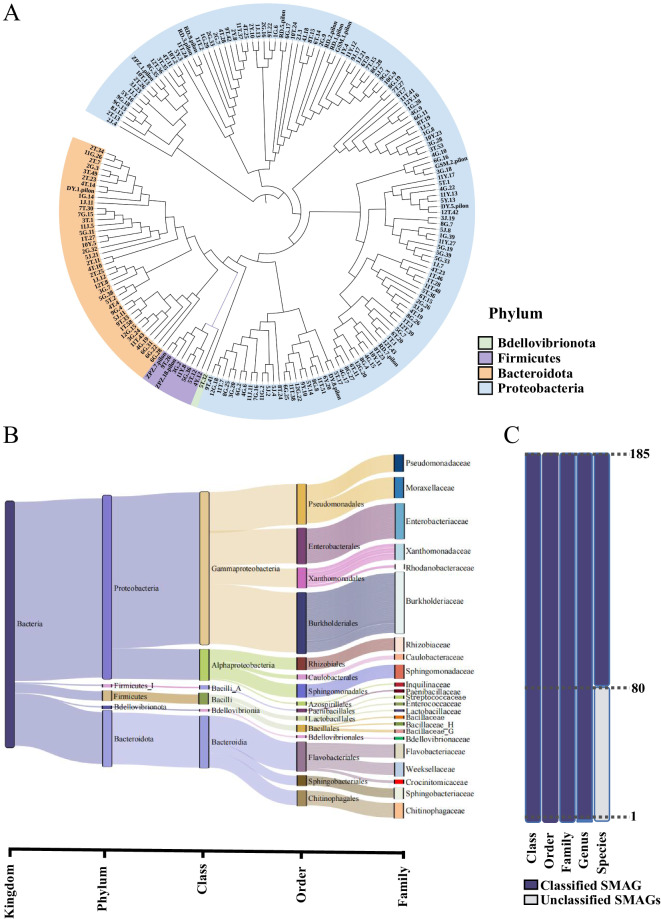
Bacterial community distribution of assembled SMAGs. (**A**) Phylogenetic tree of culturable microbes from sugarcane. Background colours in the outer circles represented microbial phyla. (**B**) Mulberry diagram showing the distribution of 185 SMAGs at different taxonomic levels. (**C**) Number of MAGs at each taxonomic level by GTDB-TK classification.

These SMAGs consisted of four phylum levels, namely *Proteobacteria* (n = 137), *Bacteroidota* (n = 38), *Firmicutes* (n = 8), and *Bdellovibrionota* (n = 1). The phylogenetic tree constructed using SMAGs also confirmed this finding (Fig. 3A). Further analysis showed that all 105 of the 185 SMAGs were identified at the species level (Fig. 3B), and 80 (43.22%) did not match the reference genome in the GTDB, and thus represented species or strains without genome sequenced before (Fig. 3C).

### Complete or near complete genomes for sugarcane culturable microbes

**Fig. 4:**
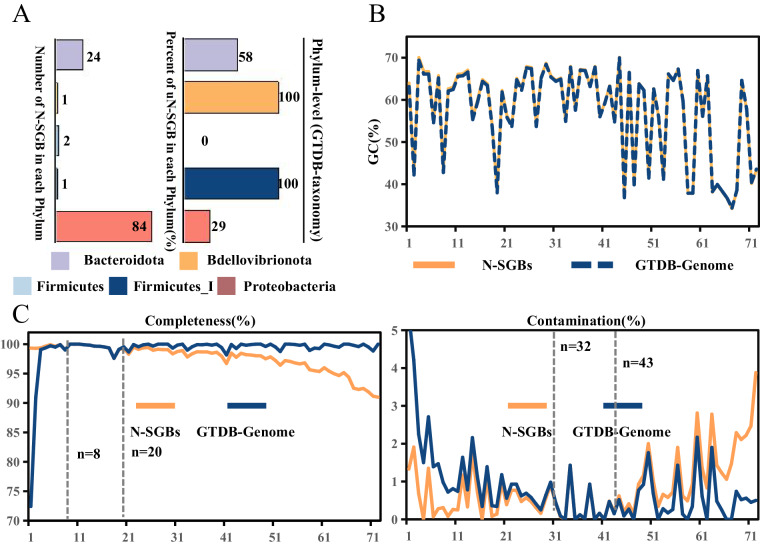
Overview of complete or near-complete genomes. (**A**) Number of complete or nearly complete species-level genomes bins (N-SGBs) and percentage of unknown N-SGBs (uN-SGBs) in each phylum. (**B)** Distribution of GC content in N-SGBs and corresponding reference genomes, with the horizontal coordinates indicating the cumulative number of genomes and the vertical coordinates showing the GC content, the yellow solid line marking N-SGBs with reference genomes, and the blue dashed line indicating reference genomes. (**C**) Distribution of completeness and contamination N-SGBs and corresponding reference genomes, with the horizontal coordinates indicating the cumulative number of genomes and the vertical coordinates indicating the completeness and contamination values, respectively, and the yellow solid line marking N-SMAGs with reference genomes and the blue solid line indicating the reference genomes.

From high-quality species-level genome bins (SGBs, >90% completeness and <5% contamination), we obtained 112 complete or near-complete species-level genomes bins (N-SGBs), of which 108 were assembled using Illumina sequencing data, and four using Nanopore sequencing data (Supplementary Data 6). *Bacteroidota* (21.4%) and *Proteobacteria* (75.0%) accounted for 96.4% of the 112 N-SGBs (Fig. 4A). For the 73 N-SGBs with reference genomes in the GTDB database, significant similarity was observed in terms of GC content between our assembly and the reference genomes (Fig. 4B). These N-SGBs showed improved genome quality in terms of completeness and contamination (Fig. 4C). Notably, the completeness of 8 genomes we assembled was 0.02% to 26.92% higher, and 31 of our assembled genomes had contamination rates 0.02% to 4.05% lower than their respective reference genomes from GTDB database (Supplementary Data 7). For the 39 unknown N-SGBs (uN-SGBs), they were widely distributed in 26 different genera (Fig. 4A, Supplementary Data 8). Two uN-SGBs contained a cluster of conserved nitrogen-fixing genes in typical nitrogen-fixing bacteria (Supplementary Data 8).

Illumina sequencing data typically results in fragmented assembled contigs in the metagenome assembly of microbial genomes. In contrast, Nanopore sequencing of long reads can be used to assemble near-complete cyclic MAGs (cMAGs), and our results confirmed this conclusion. Four Nanopore sequencing data genomes were assembled into single scaffold cyclic MAGs (cMAGs) with an average completeness of 97.50% and contamination of 0.82% (Supplementary Data 9). Among the four cMAGs, the contamination of SMAGNO90 and SMAGNO131 was 0. All the four cMAGs contained complete bacterial genome information, including 16 S, 5 S, and 23SrRNA genes and 18 tRNAs (Supplementary Data 9), and fulfilled the MINMAG criteria for “high-quality” MAGs set by the Genome Standards Consortium. 16 S rRNA genes were predicted using RNAmmer (v.1.2 http://www.cbs.dtu.dk/services/RNAmmer/)^55^. The cMAGs were annotated using the Bakta web tool (https://bakta.computational.bio), including the prediction of coding sequences (CDS), tRNAs and rRNAs. To understand the function of cMAGs, the predicted CDS was compared with the eggNOG database^56^ and KEGG database^57^ using DIAMOND (v.0.9.9.110 http://github.com/ bbuchfink/ diamond)^58^.

### Gene catalogue construction, taxonomic annotation and abundance analysis

Gene prediction by Prodigal (v.2.6 https://github.com/hyattpd/Prodigal)^59^ on contigs assembled from Illumina reads and Nanopore reads yielded 22.17 M open reading frames (ORFs). ORFs less than 100 bp in length were discarded, and then clustered to construct an initial non-redundant gene catalogue using CD-HIT-EST (v.4.5.8 https://github.com/weizhongli/cdhit)^60^. The longest ORF from each group was selected as a representative of that group. After clustering at 95% nucleotide sequence identity, we obtained a non-redundant gene catalogue of sugarcane culturable microbes (GCSCMs) containing 7,771,501 non-redundant genes. High-quality reads from Illumina sequencing of each sample were aligned to the gene catalogue using BWA-MEM (v.0.7.17), and gene abundance was calculated in transcripts per million (TPM)^61^ and corrected for variations in gene lengths and mapped read segments for each sample.

**Fig. 5:**
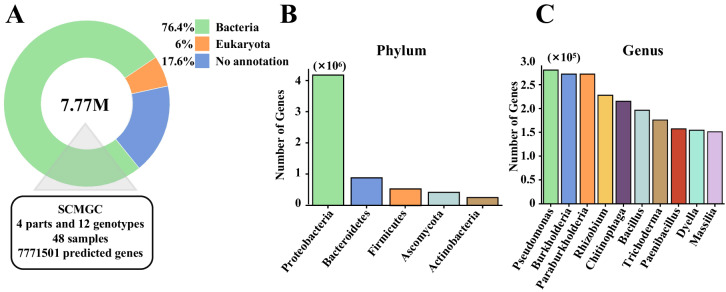
Species classification and functional annotation of the GCSCMs. (**A**) Species Taxonomic classification of the GCSCMs. (**B,C**) Categorization of gene catalogues at the phylum and genus level. Horizontal coordinates indicated the different phylum (genus) levels, and vertical coordinates indicated the total number of genes annotated to each phylum (genus) level.

ORFs were translated into protein sequences, and compared with the NR_euk database^62^ to determine the species classification information of the genes using Kaiju (https://github.com/bioinformatics-centre/kaiju). 82.4% of the genes in the GCSCMs were derived from bacteria, archaea, eukaryotes, and viruses (Fig. 5A, Supplementary Data 10), and these genes were annotated to 151 phyla, 3465 genera, and 24,084 species (Supplementary Data 11). The dominant phyla were *Proteobacteria* (65%), *Bacteroidetes* (14%), *Firmicutes* (8%), *Ascomycota* (6%), and *Actinobacteria* (3%) (Fig. 5B). Among the 3465 identified genera, the top 10 in terms of number of genes were *Pseudomonas*, *Burkholderia*, *Paraburkholderia*, *Rhizobium*, *Chitinophaga*, *Bacillus*, *Trichoderma*, *Paenibacillus*, *Dyella*, and *Massilia* (Fig. 5C).

## Data Records

The microbial genes of this study were submitted to the National Center for Biological Information (CNCB https://www.cncb.ac.cn/), China, with the BioProject accession (PRJCA019660). Gene catalogues (GCSCMs) of sugarcane culturable microbes were provided herein (https://ngdc.cncb.ac.cn/omix/release/OMIX004891)^36^. The metagenome sequencing data, and assembled genomes of this study were submitted to the National Center for Biotechnology Information, USA, with the BioProject accession (PRJNA1096928). Raw data were provided herein (NCBI Sequence Arch https://identifiers.org/ncbi/insdc.sra:SRP500217)^41^; Metagenome-assembled genomes of sugarcane culturable microbes (SMAGs) were provided herein (NCBI GenBank https://identifiers.org/ncbi/insdc:JBCNJQ000000000-JBCNNK000000000)^37,38^. Culturable bacterial single scaffold cyclic MAGs (cMAGs) from sugarcane were provided herein (NCBI GenBank https://identifiers.org/ncbi/insdc:JBCNJQ000-000000-JBCNNK000000000)^37,38^.

## Technical Validation

The completeness and contamination of the above 718 MAGs were estimated based on the lineage_wf workflow using CheckM (v.1.0.7 https://github.com/Ecogenomics/CheckM), which generated 185 SMAGs that met or exceeded moderate quality thresholds (≥50% completeness and ≤10% contamination), with quality scores for each MAG calculated based on completeness - 5*contamination. Gene prediction by Prodiga on contigs assembled from Illumina reads and Nanopore reads yielded 22.17 M open reading frames (ORFs).

### Supplementary information

Supplementary Data 1

Supplementary Data 2

Supplementary Data 3

Supplementary Data 4

Supplementary Data 5

Supplementary Data 6

Supplementary Data 7

Supplementary Data 8

Supplementary Data 9

Supplementary Data 10

Supplementary Data 11

## Data Availability

All the tools mentioned in the data analysis used in this study were publicly available and the sources and versions of the analytical programs and codes were indicated in the Materials and Methods. No custom code or pipelines were generated in this manuscript.
